# Association of Hepatic Lipase -514T Allele with Coronary Artery Disease and Ankle-Brachial Index, Dependence on the Lipoprotein Phenotype: The GENES Study

**DOI:** 10.1371/journal.pone.0067805

**Published:** 2013-07-09

**Authors:** Céline Verdier, Jean-Bernard Ruidavets, Vanina Bongard, Dorota Taraszkiewicz, Laurent O. Martinez, Meyer Elbaz, Jean Ferrières, Bertrand Perret

**Affiliations:** 1 CHU Toulouse, Department of Biochemistry, Toulouse, France; 2 INSERM UMR 1048, Institut des Maladies Métaboliques et Cardiovasculaires de Toulouse, Université de Toulouse III, Toulouse, France; 3 CHU Toulouse, Department of Epidemiology, Toulouse, France; 4 INSERM UMR 1027, –Epidémiologie et Analyses en Santé publique, Université de Toulouse III, Toulouse, France,; 5 CHU Toulouse, Department of Cardiology, Toulouse, France; Wageningen University, The Netherlands

## Abstract

**Objectives:**

Relationship between hepatic lipase (*LIPC*) polymorphism and coronary artery disease (CAD) has often led to contradictory results. We studied this relation by genotyping rs1800588 in the *LIPC* promoter in a case-control study on CAD (the GENES study). We also investigated the relationship between this polymorphism and the ankle-brachial index (ABI), which is predictive of atherosclerosis progression and complications in patients at high cardiovascular risk.

**Methods:**

557 men aged 45–74 with stable coronary artery disease and 560 paired controls were genotyped for rs1800588. Medical data, clinical examination including determination of ABI and biological measurements related to cardiovascular risk factors enabled multivariate analyses and multiple adjustments.

**Results:**

CAD cases showed a higher T-allele frequency than controls (0.246 *vs* 0.192, p = 0.003). An interaction has been found between *LIPC* polymorphism and triglycerides (TG) levels regarding risk of CAD: TT-homozigosity was associated with an Odds ratio (OR) of 6.4 (CI: 1.8–22.3) when TG were below 1.5 g/L, but no association was found at higher TG levels (OR = 1.34, CI: 0.3–5.9). The distribution of *LIPC* genotypes was compared between CAD patients with normal or abnormal ABI and impact of *LIPC* polymorphism on ABI was determined. Following multiple adjustments, association of the T-allele with pejorative ABI (<0.90) was significant for heterozygotes and for all T-carriers (OR = 1.55, CI: 1.07–2.25).

**Conclusion:**

The -514T *LIPC* allele is associated with CAD under normotriglyceridemic conditions and constitutes an independent determinant of pejorative ABI in coronary patients.

## Introduction

HDL-cholesterol (HDL-C) is established as inversely related to cardiovascular risk. Among candidate genes, *LIPC* is one displaying the strongest impact on HDL-cholesterol (HDL-C) concentrations [1], [2] and would account for over 50% of HDL-C genetic variability in humans [3]. Most of it depends on a common polymorphism of the *LIPC* promoter, of which the C-514T is a signature. Variant allele confers a decreased expression and activity of LIPC [4]. Its impact on atherogenesis is still a matter of debate [5]: a pro-atherogenic role of LIPC is hypothesized in light of its functions in the catabolism of HDL and in the formation of small and dense LDL. Conversely, other functions of LIPC might be anti-atherogenic, like its effects on HDL liver uptake, the last step of reverse cholesterol transport, and its ability to complete the lipolysis of TG-rich lipoproteins [6]. Impact of *LIPC* variation on clinical atherosclerosis is also controversial [7]–[10]. Recent reviews suggest that the impact of *LIPC* on CAD would be dependent on the underlying lipoprotein phenotype [5], [11]. Low HDL is a frequent abnormality among CAD patients [12], often associated with hypertriglyceridemia as components of metabolic syndrome occurring in a climate of insulin resistance. In the present study, we have re-evaluated the relationship between *LIPC* polymorphism and the atherosclerotic disease, comparing stable CAD-patients to paired control subjects taken from the general population. We have also considered the ankle-brachial index (ABI), a clinical non-invasive index reflecting the progression of the atherosclerotic disease in CAD patients. Indeed, ABI has been considered as a prognosis marker of further complications in patients at high cardiovascular risk [13], [14]. In this respect, a recent study from our group has shown that abnormal ABI is predictive of mortality among CAD patients [15]. Our present observations support a deleterious impact of the *LIPC* -514T variant on this index of atherosclerosis progression in CAD patients.

## Methods

### Study Population

The “Génétique et Environnement en Europe du Sud” (GENES) study is a case-control study designed to assess the role of genetic, biological and environmental determinants in the occurrence of CAD. As previously described [15], cases were stable male CAD patients living in the Toulouse area (South-western France), aged between 45 and 74 and recruited from 2001 to 2004 after admission in the department of cardiology of the Toulouse University Hospital. They were admitted for cardiovascular examination, in the context of evaluation and management of their CAD. Stable CAD was defined by a previous history of acute coronary syndrome, a previous history of coronary artery revascularization, a documented myocardial ischemia, a stable angina, or the presence at the coronary angiography of a coronary stenosis ≥50%. Patients who had presented a recent acute coronary episode were considered as not stable and were not included in the study. During the same period, male controls, aged 45–74 years, were selected from the general population using electoral rolls. Stratification by 10-age group was employed to approximately match the age distribution between controls and cases. Information was provided about the objectives of the study, and informed consent was signed. Controls and cases underwent medical examination in the same health centre and during the same period, including clinical and anthropometric measurements and completion of a questionnaire. Blood samples were taken. In the present analysis, we took into account approximately the first 600 patients and 600 control subjects, in whom genotyping for *LIPC* genes was performed. The whole study protocol has been conducted according to the principles of the Declaration of Helsinki. The protocol was endorsed by the Scientific Council of the Toulouse University Hospital. It was approved by the “Comité Consultatif pour la Protection des Personnes se prêtant à la Recherche Biomédicale” (Advisory Committee regarding protection of persons involved in medical investigation, Comité Toulouse/Sud-Ouest #1) file number 1-99-48. The biological sample collection was declared to the French Ministry of Research and to the Regional Health Agency under number DC-2008-403 #1. Information was provided about the objectives of the study, and informed consent was signed.

### Data Collection

Age, socioeconomic variables and information on cardiovascular risk factors were collected through standardized face-to-face interviews, performed by a single physician. Smoking status was classified as current smokers, former smokers having quitted tobacco for more than 3 months and non-smokers. Alcohol consumption was assessed using a typical week pattern. The total amount of alcohol consumed was calculated as the sum of different types of drinks allowing categorization into three levels: abstainers, moderate alcohol consumption (1–39 g/day) and heavy consumption ≥40 g/day. Physical activity was investigated through a standardized questionnaire and categorized into two levels: no or moderate physical activity during 20 min less than once a week, and intense physical activity ≥20 min each session ≥ twice a week. Presence of dyslipidemia, diabetes or hypertension was assessed from the subjects’ current treatments. In patients, medications at discharge were also considered. Anthropometrical measurements included waist circumference, height, body weight and body mass index (BMI) was calculated (kg/m^2^). Blood pressure and resting heart rate were measured after ≥5 min rest with an automatic sphygmomanometer. Two measurements were performed and average values were recorded. Metabolic syndrome was defined according to the NCEP-ATPIII [16] and insulin resistance was estimated by the Homeostasis Model Assessment of Insulin Resistance (HOMA-IR). ABI was evaluated as previously described [15]. The lower limb blood pressure was determined from the right and left posterior tibial arteries with the patient in supine position. When the posterior tibial artery blood pressure was not measurable, the *dorsalis pedis* artery was used. The systolic blood pressure was detected with a hand-held Doppler probe. ABI was calculated for each lower limb by dividing the ankle systolic blood pressure by the average of the two measurements performed on the arm. For each patient, the lowest ABI recorded in the 2 ankles was kept for further analysis. An ABI <0.9 was considered abnormal.

### Biological Measurements

Blood was collected after an overnight fast. Glycaemia, triglycerides, total cholesterol and HDL-cholesterol were assayed with enzymatic reagents on an automated analyser (Hitachi 912, Roche diagnostics). LDL-cholesterol was calculated using the Friedwald formula. CRP and Gamma-GT were also analysed with an automated analyser (Hitachi, Roche-Diagnostics). Insulin measurements were done using an immunometric assay (Advia Centaur, Siemens); adiponectin was measured by an ELISA technique (R&D systems). ApoA-I, apo-B and lipoprotein (Lp) (a) were determined by first order precipitation in an automated analyser (Hitachi, Roche-Diagnostics). Total apoE and apoC-III were measured by electro-immunoassay (Sebia, France). LpA-I was also determined by electro-immunoassay (Sebia).

### Genetic Polymorphism

Genomic DNA was isolated from EDTA-treated blood, using silica columns (Macherey-Nagel). Genotyping at position -514 of the *LIPC* gene (rs1800588) was performed with polymerase chain reaction (PCR) amplification. DNA was amplified using primers 5′-AAG AAG TGT GTT TAC TCT AGG ATC A-3′ (sense) and 5′-GGT GGC TTC CAC GTG GCT GCC TAA G-3′ (anti-sense) with an annealing temperature of 58°C. The C-514T substitution introduces a *NlaIII* endonuclease restriction site. PCR products were incubated for 16h with *NlaIII* (New England Biolabs). Restriction fragments were separated by electrophoresis on an ethidium bromide-pre-stained 2% Agarose gel. The wild-type PCR product does not contain a *NlaIII* restriction site and yields a 299 bp band. Heterozygotes display bands of 299, 215 and 84 bp whereas homozygotes show bands of 215 and 84 bp.

### Statistical Analyses

In descriptive tables, data are presented as percentages and numbers for qualitative variables, and as means with standard deviations for quantitative ones. The Chi-squared test was used to compare the distribution of qualitative variables between CAD cases and controls and, among CAD patients, between subjects with ABI <0.9 and with ABI ≥0.9. The mean values of quantitative variables were compared by Student’s *t-test*. Shapiro-Wilks was used to test the normality of distribution of residuals and Levene’s test to test the homogeneity of variances. When basic assumptions of Student’s *t-test* were not satisfied, data were logarithmically transformed or subjected to a Wilcoxon-Mann-Whitney test. Two-way analysis of variance was used to test the association of lipids, lipoproteins and biological markers of glucose regulation with the CAD status and *LIPC* genotypes. Interactions between the case-control design, *LIPC* genotypes and all the studied variables were verified.

Multivariate stepwise logistic regression analysis was used to determine independent associations between the CAD status and key exposures (*LIPC*), adjusting for covariates at various levels. The model was built using stepwise regression of the candidate variables with *p* values ≤0.10 to enter the model and ≤0.05 to be maintained. In addition, the multivariate model was adjusted for diabetes, hypertension, dyslipidemia, smoking, alcohol consumption, physical activity, CRP, HDLc, Lp(a) and triglycerides. A further adjustment on ABI was done. Because a significant interaction of *LIPC* with triglycerides was found (p<0.10), a stratification analysis was carried out according to different cut-offs of plasma TG. Data are illustrated for the cut-off between the 2^nd^ and 3^rd^ tertiles (1.5 g/L), corresponding to the limit between normo- and hyper-triglyceridemic subjects. Similarly, to determine independent associations between ABI status and *LIPC*, multivariate stepwise logistic regression analysis was performed adjusting for various covariates. The multivariate model was adjusted for diabetes, smoking, physical activity and heart rate.

The performance of final models was evaluated by analysis of the area under the receiver operating characteristic (ROC) curve (AUC). The internal validity of the prediction models was evaluated by bootstrapping [17], [18], using 100 random re-samplings of equal size from the complete data set. Performances (AUC) in the bootstrap samples and in the original sample are given in legends to tables.

Analyses were two-tailed and p<0.05 was considered to be significant. The false discovery rate (FDR) method was used to correct for multiple comparisons in subgroup analyses (according to ABI values). Statistical interactions were tested and considered as significant at p≤0.20. Computation was carried out with SAS software, version 9.2 (SAS Institute, Cary, IL, USA).

## Results

### Characteristics of Cases and Controls

In the GENES study, genotyping of different loci affecting metabolism of plasma lipoproteins was carried out among 600 pairs of CAD patients and control subjects. Regarding *LIPC* variants, complete data were available for 560 controls and 557 cases (Table 1). Treatments for major cardiovascular risk factors, including diabetes, dyslipidemia, hypertension, and smoking habits were much more frequent in cases than in controls. Conversely, physical activity was less frequent in cases. Cases displayed characteristics of metabolic syndrome, with elevated glycaemia and triglycerides and low HDL concentrations. Levels of pro-atherogenic apo CIII- and apo E-containing particles derived from the metabolism TG-rich lipoproteins, were increased in CAD cases. Signs of insulin resistance were also evident in cases, such as elevated waist circumference, high insulin levels and HOMA index. Also, adiponectin, an insulin sensitizing adipokine, was significantly lower in cases than in controls. Concordantly, prevalence of metabolic syndrome according to the NCEP-ATPIII definition was 50% among cases, compared to 17% in controls. Cases displayed rather low levels of cholesterol and apo B, and blood pressure figures were comparable with those in controls, which might reflect the effectiveness of administered treatments. Frequencies of subjects with elevated levels of CRP (≥3 mg/l), Lp(a) (≥0.25 g/L) and GGT (≥36 IU/L) were much higher in CAD cases than in controls. Finally, a pejorative value of ABI (<0.9) was evident in 32.5% of CAD patients and in only 1.6% of controls. Distribution of *LIPC* genotypes was significantly different between controls and cases (Table 1). The calculated T-allele frequency was 0.192 in controls *versus* 0.246 in CAD cases (p = 0.003, not shown). Both population samples did not diverge from the Hardy-Weinberg equilibrium (p = 0.88).

**Table 1:** Characteristics according to case-control design.

	Cases	Controls	p
	n = 557	n = 560	
*LIPC*			0.001
CC	57.8% (322)	63.6% (356)	
CT	35.2% (196)	34.3% (192)	
TT	7.0% (39)	2.1% (12)	
Age (years)	60.3 (8.1)	58.7 (8.3)	0.002
BMI (kg/m^2^)	27.3 (4.0)	26.8 (3.4)	0.05**
Waist circumference (cm)	99.0 (10.7)	94.8 (9.6)	0.001
Systolic blood pressure (mmHg)	138 (20)	137 (15)	0.14**
Heart rate (beats/mn)	63.9 (12.2)	63.3 (9.2)	0.31**
Glycaemia (g/L)	1.10 (0.39)	0.98 (0.18)	0.003**
Insulin (IU/L)	16.1 (23.8)	9.9 (7.2)	0.001*
HOMAI-IR	4.7 (10.2)	2.5 (2.3)	0.001*
Adiponectin (mg/L)	5.8 (4.5)	7.0 (4.6)	0.001
TC (g/L)	2.02 (0.42)	2.25 (0.38)	0.001
HDLc (g/L)	0.43 (0.12)	0.56 (0.13)	0.001
LDLc (g/L)	1.26 (0.37)	1.46 (0.33)	0.001
Apo A-1 (g/L)	1.23 (0.23)	1.52 (0.24)	0.001
Apo B (g/L)	1.05 (0.24)	1.10 (0.22)	0.001
TG (g/L)	1.68 (0.94)	1.19 (0.78)	0.001*
Apo C3 (g/L)	33.5 (13.8)	30.8 (13.0)	0.001
Apo E (g/L)	102.8 (61.0)	74.7 (47.2)	0.001*
LpA1 (g/L)	0.46 (0.14)	0.56 (0.18)	0.001*
Lp(a) ≥0.25 g/L (median)	61.0% (340)	40.2% (225)	0.001
CRP≥3 mg/L (median)	75.4% (420)	31.3% (175)	0.001
GGT ≥36 IU/L (median)	58.2% (324)	44.1% (247)	0.001
Metabolic syndrome (NCEP-ATPIII)	50.4% (278)	16.8% (94)	0.001
Diabetes	24.1% (134)	4.6% (26)	0.001
Dyslipidemia	60.7% (338)	23.4% (131)	0.001
Hypertension	44.7% (249)	18.7% (105)	0.001
Former and current smokers	81.0% (452)	65.0% (364)	0.001
Alcohol (1–39 g/day)	53.9% (300)	66.1% (370)	0.001
Intense physical activity	11.5% (64)	38.7% (217)	0.001
Ankle-brachial index <0.9	32.5% (181)	1.6% (9)	0.001

Mean (standard deviation) or % (n).

* Analyses were performed on log transformed data.

** Kruskal-Wallis test.

### Metabolic Characteristics According to *LIPC* Genotypes

Table 2 shows distribution of *LIPC* genotypes in control subjects and CAD cases and their association with various metabolic markers. Homozygotes for the T-allele of *LIPC* promoter were 3-fold more numerous among cases than in controls (39 versus 12). The *LIPC* T-allele was associated with increased levels of cholesterol and apo B, of TG and apo C-III, with an allele-dose effect for the latter. TT-homozygotes also displayed elevated apo A-I (p = 0.07), reaching significance in the control subpopulation (p = 0.04, not shown). No significant association was found between *LIPC* genotype and markers of insulin resistance, occurrence of metabolic syndrome (Table 2) or with anthropometric variables (not shown).

**Table 2:** Lipids, lipoproteins and metabolic markers according to *LIPC* genotypes and case-control design.

		Cases			Controls			
							p value forcase-controleffect	p value for*LIPC*effect
	CC	CT	TT	CC	CT	TT		
Numbers of subjects	322	196	39	356	192	12		
TC (g/L)	1.99 (0.43)	2.05 (0.38)	2.08 (0.52)	2.22 (0.35)	2.29 (0.41)	2.35 (0.51)	0.001	0.03
HDLc (g/L)	0.43 (0.12)	0.43 (0.14)	0.44 (0.10)	0.55 (0.12)	0.56 (0.14)	0.62 (0.12)	0.001	0.31
LDLc (g/L)	1.25 (0.37)	1.27 (0.34)	1.31 (0.48)	1.45 (0.32)	1.47 (0.35)	1.49 (0.42)	0.001	0.40
TG (g/L)*	1.62 (0.96)	1.77 (0.91)	1.73 (0.91)	1.13 (0.61)	1.30 (1.02)	1.33 (0.74)	0.001	0.02
Apo A-1 (g/L)	1.22 (0.21)	1.23 (0.25)	1.27 (0.18)	1.51 (0.23)	1.52 (0.25)	1.68 (0.21)	0.001	0.07
Apo B (g/L)	1.04 (0.23)	1.07 (0.22)	1.10 (0.32)	1.09 (0.21)	1.13 (0.22)	1.13 (0.28)	0.001	0.04
Apo E (g/L)*	99.6 (59.3)	108.5 (61.4)	100.7 (71.9)	72.3 (42.3)	79.7 (55.2)	67.1 (43.8)	0.001	0.12
Apo C3 (g/L)*	32.3 (12.5)	34.3 (12.8)	39.6 (23.5)	30.2 (11.9)	31.5 (14.7)	35.8 (16.8)	0.001	0.009
Glycaemia (g/L)*	1.11 (0.41)	1.06 (0.35)	1.10 (0.45)	0.97 (0.17)	0.99 (0.20)	1.00 (0.19)	0.001	0.86
Insulin (IU/L)*	16.1 (21.3)	14.9 (23.6)	23.1 (38.5)	9.5 (6.8)	10.5 (7.8)	10.8 (6.9)	0.001	0.37
HOMA-IR*	4.9 (11.8)	4.1 (6.5)	6.5 (10.9)	2.4 (2.1)	2.7 (2.7)	2.8 (2.3)	0.001	0.45
Adiponectin (mg/L)	5.8 (4.2)	6.0 (5.1)	6.0 (4.6)	7.2 (4.9)	6.6 (3.9)	4.8 (3.3)	0.001	0.62
Metabolic syndrome (NCEP-ATPIII)	52.8%	46.9%	48.7%	16.3%	17.7%	16.6%	0.001	0.72

* Analyses were performed on log transformed data.

### Association of *LIPC* Polymorphism with the Status of CAD Case

Considering the T-allele higher prevalence in CAD cases than in controls, we investigated the impact of *LIPC* polymorphism on the risk to be a case (Table 3). In univariate analysis, TT-homozygotes presented an OR of 3.62 (CI: 1.87–7.06) of being a CAD-case, while OR for CT-heterozygotes was non-significant. Then, multivariate analyses were performed taking into account major cardiovascular risk factors and parameters having proved to be different between cases and controls. Even after multiple adjustments on those established factors or markers, TT-homozygosity conferred an increased risk of ischemic heart disease (OR = 3.41, CI: 1.22–9.59). Interestingly, a statistic interaction was evidenced between *LIPC* polymorphism and TG levels regarding the risk of CAD (Chi2 = 4.61, *p* = 0.10). Thus impact of *LIPC* polymorphism was studied at different cut-offs of triglyceride distribution in the whole population. Table 3 illustrates association of *LIPC* genotype to CAD from both sides of the 2^nd^–3^rd^ tertiles cut-off, at 1.5 g/L, corresponding to the upper limit of normal values. In normotriglyceridemic subjects, TT-homozigosity was strongly associated to CAD, with an OR of 6.4 (CI: 1.8–22.3). This association was no longer observed in hypertriglyceridemic conditions (≥1.5 g/L): OR was 1.34, (CI: 0.3–5.9). The analysis was resumed using different cut-offs for plasma triglycerides, (the 50^th^ and 75^th^ percentiles, corresponding to 1.22 and 1.75 g/L) leading to similar observations: significant association of -514T homozigosity to CAD at low triglyceridemias and no more association at highest TG levels.

**Table 3:** Risk for CAD according to *LIPC* genotypes.

				TG*LIPC Interaction			TG <1.50 g/L			TG ≥1.50 g/L	
	OR	95% CI	p	CHI2	p	OR	95% CI	p	OR	95% CI	p
Unadjusted											
CT *vs* CC	1.12	0.87–1.44	0.36								
TT *vs* CC	3.62	1.87–7.06	0.001								
CT and TT *vs* CC	1.27	0.99–1.62	0.06								
Adjusted^1^				4.61	0.10						
CT *vs* CC	0.93	0.65–1.33	0.69			0.93	0.58–1.49	0.77	0.79	0.43–1.45	0.46
TT *vs* CC	3.41	1.22–9.59	0.02^2^			6.37	1.82–22.3	0.004^3^	1.34	0.30–5.93	0.70
CT and TT *vs* CC	1.04	0.73–1.47	0.84								

1 Adjusted for diabetes, hypertension, dyslipidemia, smoking, alcohol consumption, physical activity, CRP, HDLc, Lp(a), triglycerides and ABI.

2 Area under the curve (AUC) for adjusted model: 0.91; corrected AUC after bootstrap validation: 0.90.

3 Area under the curve (AUC for adjusted model: 0.92; corrected AUC after bootstrap validation: 0.91.

### Association of *LIPC* Polymorphism with a Pejorative ABI in CAD-cases

Because a high proportion (32.5%) of CAD-cases displayed a pejorative ABI (<0.9), the characteristics of those patients were compared with those cases having ABI above 0.9 (Table 4). Among the major cardiovascular risk factors, smoking habits, lack of physical activity, presence of diabetes, increased systolic blood pressure and heart rate were strongly associated with low ABI. None of the investigated metabolic parameters was significantly different according to ABI, except for a trend to higher adiponectin in patients with a pejorative index (p = 0.07). The distribution of *LIPC* genotypes was compared between patients with normal or abnormal ABI. Clearly, T-carriers were much more frequent among patients with low ABI, with an allele frequency of 0.30 as compared to 0.22 in patients with normal ABI (p = 0.03, not shown). Impact of *LIPC* polymorphism on ABI was further determined (Table 5). Unadjusted analyses indicated that the OR for having a low index was 1.55 (CI: 1.08–2.22) for all T-carriers. Following multiple adjustments on cardiovascular risk factors and markers, association of the T-allele with a pejorative ABI remained significant for heterozygotes and for all T-carriers (OR = 1.55, CI: 1.07–2.25). Results for TT-homozygotes failed to be significant, possibly due to lack of statistical power.

**Table 4:** Characteristics according to ankle-brachial index status in CAD patients.

	<0.9	≥0.9	p
	181 (32.5)	376 (67.5)	
*LIPC*			0.03
CC	50.3% (91)	61.4% (231)	
CT	40.3% (73)	32.7% (123)	
TT	9.4% (17)	5.9% (22)	
Age (years)	60.9 (7.9)	60.0 (8.1)	0.19
TC (g/L)	2.05 (0.43)	2.01 (0.42)	0.32
HDLc (g/L)	0.44 (0.13)	0.43 (0.12)	0.27
LDLc (g/L)	1.28 (0.38)	1.25 (0.36)	0.35
TG (g/L)	1.69 (0.94)	1.67 (0.96)	0.80
ApoA1 (g/L)	1.23 (0.22)	1.23 (0.23)	0.84
ApoB (g/L)	1.07 (0.25)	1.05 (0.23)	0.32
Lp(a) (g/L)*	0.49 (0.49)	0.48 (0.44)	0.77
ApoE (mg/L)	99.7 (59.2)	104.2 (61.9)	0.41
ApoC3 (mg/L)	33.4 (13.4)	33.5 (13.9)	0.98
LpA1 (g/L)	0.46 (0.16)	0.45 (0.14)	0.36
CRP (mg/L)	16.6 (23.9)	13.7 (24.4)	0.20
GGT (IU/L)*	68.0 (74.6)	59.7 (59.2)	0.37
Glycaemia (g/L)	1.10 (0.43)	1.09 (0.38)	0.91
Insulin (IU/L)*	17.7 (26.3)	15.4 (22.4)	0.16
HOMA-IR*	5.6 (15.1)	4.3 (6.6)	0.29
Adiponectin (mg/L)	6.4 (4.8)	5.6 (4.4)	0.07
BMI (kg/m^2^)	27.1 (3.9)	27.5 (4.0)	0.26
Waist circumference (cm)	98.9 (11.1)	99.1 (10.5)	0.87
Systolic blood pressure (mmHg)	140.7 (18.9)	136.6 (20.3)	0.03
Heart rate (beats/mn)	66.0 (13.4)	64.0 (11.5)	0.007
Metabolic syndrome (NCEP-ATPIII)	48.6% (89)	50.9% (191)	0.75
Diabetes	30.4% (55)	21.0% (79)	0.02
Dyslipidemia	62.4% (113)	59.8% (225)	0.56
Hypertension	49.2% (89)	42.6% (160)	0.15
Never smoker	12.2% (22)	22.1% (83)	0.005
Alcohol (1–39 g/day)	50.3% (91)	44.2% (166)	0.18
Intense physical activity	7.2% (13)	13.6% (51)	0.03
			

* Log transformed data.

Mean (standard deviation) or % (n).

**Table 5:** Risk for ankle-brachial index <0.90 according to *LIPC* genotypes in CAD patients.

	OR	95% CI	p^3^
Unadjusted			
CT *vs* CC	1.50	1.03–2.19	0.03
TT *vs* CC	1.97	0.92–3.65	0.09
CT and TT *vs* CC	1.55	1.08–2.22	0.02
Adjusted**^1^** ^–**2**^			
CT *vs* CC	1.52	1.03–2.25	0.036
TT *vs* CC	1.71	0.84–3.48	0.14
CT and TT *vs* CC	1.55	1.07–2.25	0.036

1 Adjusted for smoking, diabetes, physical activity and heart rate.

All interactions between ankle-brachial index, *LIPC* genotypes and variables of adjustment were non-significant.

2 Area under the curve (AUC) for adjusted model: 0.73, corrected AUC after bootstrap validation: 0.72.

3 False discovery rate method was used to correct for multiple comparisons for subgroup analyses. Corrected p values are shown.

## Discussion

Epidemiological studies have demonstrated the negative association between HDL and cardiovascular diseases. However, relevance of HDL for risk assessment has been recently questioned, as several mechanisms leading to elevated HDL levels do not unequivocally lead to cardiovascular risk protection [10], [11], [19]. Herein, we have evaluated the impact of a common genetic variant of *LIPC* in a large case-control study on CAD. T-allele homozygotes were found at increased risk of CAD. Moreover, among CAD patients, T-allele was associated with a pejorative value of the ABI, an indicator of infra clinical peripheral arterial disease and a prognosis marker in CAD patients.

Several studies have clearly demonstrated that the *LIPC* T-allele is associated to a lower hepatic lipase activity and a rise in HDL [4], [20]. It has been suggested that 38% of HDL-C variability would be explained by genetic variance, of which *LIPC* would contribute for up to 53% [3]. Recently, this association between *LIPC* polymorphisms and HDL was further confirmed in large genome-wide association studies [21], [22]. In the present study, association of the -514T allele with elevated apo A-I was observed in controls (p = 0.04) but not among cases, probably because of other interfering factors, like hypertriglycerdidemia or administered treatments.

During the last decade, several clinical studies have examined the relationship between *LIPC* variants (−250 or −514) and CAD, leading to conflicting results [7]–[10], [23]. For instance, regarding preclinical atherosclerosis, the −514T allele has been recently found associated to increased carotid atherosclerosis, as documented by intima media thickness and plaques [8], whereas another study concluded to absence of relationship [7]. Regarding the risk of CAD, studies concluded to no impact of *LIPC* variability while others demonstrated a negative effect of the −514T variant on CAD [9], [11]. Interestingly, in the large secondary prevention REGRESS study, it was demonstrated that patients having a combination of defective alleles for *CETP* and *LIPC*, displayed high HDL levels but also the highest progression of coronary atherosclerosis [11]. Conclusions from meta-analyses also diverge regarding association between *LIPC* −514T and CAD [10], [19]. Of note, one recent and among the largest meta-analyses dealing with SNPs affecting HDL-cholesterol found an association of *LIPC* rs1800588 with myocardial infarction [19]. Among possible reasons for such discrepancies, might be evoked differences in the study populations or in the definition of control subjects.

The present investigation has, in its design, the limitations of a cross-sectional, case – control study. However, with a clear definition of CAD cases and paired controls taken from the general population, it shows that *LIPC* T-allele is associated to both the CAD status and to ABI, a marker of atherosclerosis progression. Recently, a large Mendelian randomisation study, cited above [19], clearly demonstrated that several genetic determinants that raise HDL-cholesterol, including the parent *LIPG* coding for endothelial lipase, do not lower the cardiovascular risk. All those observations converge to suggest that genetic variations that increase HDL but impair their normal catabolism might be deleterious regarding atherosclerotic diseases.

In this study, a large proportion of patients (60%) were under cholesterol lowering therapy, explaining why LDL-cholesterol and apo B were lower in cases than in controls. However, all multivariate analyses were adjusted on lipid levels and treatment for dyslipidemia, and the −514T allele frequencies were identical when subjects under lipid lowering therapy were excluded (not shown). The cases’ metabolic profile was quite evocative of metabolic syndrome, with low HDL, increased levels of TG-rich lipoproteins and moderate hyperglycaemia. Moreover, elevated insulin and HOMA index together with low adiponectin were signs of insulin resistance. Prevalence of metabolic syndrome according to NCEP-ATPIII was 50%, 3-times as much as in control subjects. Apo C-III is a major protein of VLDL, which accumulates in conditions of insulin-resistance. Increased levels of apo C-III and apo E reflects prolonged residence of TG-rich lipoproteins and their remnants, associated with abnormal persistence of circulating free fatty acids, which favour insulin resistance. Moreover, remnants derived from TG-rich lipoproteins are pro-atherogenic, and large prospective trials in CAD-patients have demonstrated that apo C-III-containing particles are the best predictors of lesion progression [24].

Several functions of LIPC argue in favour of its anti-atherogenic roles. LIPC acts on HDL phospholipids and TG prior to HDL uptake by liver. Thus, LIPC is a key partner in the final step of reverse cholesterol transport. Another impact of LIPC is the degradation of IDL and remnant particles derived from TG-rich lipoproteins, known to be highly atherogenic. On the other hand, a pro-atherogenic role of LIPC in dysmetabolic conditions has been postulated [20]. In hypertriglyceridemic and insulin resistant situations, with HDL and LDL enriched in triglycerides, LIPC would promote the formation of small and dense LDL and accelerate HDL catabolism [6], [20]. Thus, it was suggested that the effects of LIPC on atherogenesis would depend on the underlying lipoprotein phenotype [5], [20]. Our present observations firmly support this concept since the impact of *LIPC* variant on the CAD status was dependent on the plasma triglyceride concentration. Under normotriglyceridemic conditions (<1.5 g/L of plasma TG), the defective *LIPC* variant displayed a strong association with CAD, probably due to impaired reverse cholesterol transport. In hypertriglyceridemic conditions, association of *LIPC* variant to CAD was lost. This suggests that defective *LIPC* would no longer be pro-atherogenic, as, on the opposite, it might counteract formation of small and dense LDL and slow down catabolism of apo A-I, helping to maintain HDL levels. Concordant with those observations, it was formerly reported that in dyslipidemic patients, *LIPC* polymorphisms would predict response to lipid lowering therapy in terms of atherosclerosis regression [25].

Lower limb arterial disease (LLA) is frequent among patients with multiple cardiovascular risk factors. ABI has emerged as a non-invasive, easily implementable and reproducible tool to screen for the presence of LLA at a pre-clinical stage. A cut-off value of 0.9 could distinguish patients who need further exploration [26]. Herein, risk factors classically related to LLA (diabetes, smoking and increased blood pressure) were also those most strongly associated with low ABI. Moreover, in CAD-patients low ABI would be predictive of secondary events [13]. In the context of the GENES study follow-up, we recently demonstrated that ABI is an independent prognosis factor of all-cause mortality [15]. In the present paper, the *LIPC* T-allele appears as an independent determinant of a pejorative ABI in CAD-patients. The pro-atherogenic potential of the T-allele is reflected by its increased prevalence, from 0.19 in healthy controls to 0.22 in CAD-patients with ABI ≥0.9, and up to 0.30 in those having low ABI. A previous cross-sectional study on lower limb arterial disease has documented an impact of the −250A variant, which is in linkage disequilibrium with the −514T [27]. More recently, the same variant was found associated with ABI among diabetics [28]. The present study extends these observations to the most common −514 T in CAD-patients, in whom ABI was demonstrated to be a prognosis factor. Interestingly, while association to patent CAD was demonstrated only for T-allele homozygotes, association with pejorative ABI, a marker of infra-clinical disease progression, was evident for all T-carriers, confirming that T-allele carriage is indeed deleterious regarding atherosclerotic disease.

In conclusion, we have observed that the *LIPC*-T allele is independently associated with a clinical index of atherosclerosis progression, predictive of further complications among coronary patients.
